# Combined use of smartphone and smartband technology in the improvement of lifestyles in the adult population over 65 years: study protocol for a randomized clinical trial (EVIDENT-Age study)

**DOI:** 10.1186/s12877-019-1037-y

**Published:** 2019-01-23

**Authors:** José I. Recio-Rodríguez, Cristina Lugones-Sanchez, Cristina Agudo-Conde, Jesús González-Sánchez, Olaya Tamayo-Morales, Susana Gonzalez-Sanchez, Carmen Fernandez-Alonso, Jose A. Maderuelo-Fernandez, Sara Mora-Simon, Manuel A. Gómez-Marcos, Emiliano Rodriguez-Sanchez, Luis Garcia-Ortiz

**Affiliations:** 1 Institute of Biomedical Research of Salamanca (IBSAL), Primary Health Care Research Unit, La Alamedilla Health Center. Health Service of Castilla y León (SACyL), Primary Care Prevention and Health Promotion Research Network (REDIAPP), Salamanca, Spain; 2 Faculty of Health Sciences, University of Burgos, Burgos, Spain; 3 Department of Nursing, University of Extremadura, Plasencia, Cáceres Spain; 4 Casa del Barco Health Center, Castilla y León Health Service, Valladolid, Spain; 5 Department of Basic Psychology, Psychobiology and Behavioral Sciences Methodology, University of Salamanca, Salamanca, Spain; 6 Department of Medicine, University of Salamanca, Salamanca, Spain; 7 Department of Biomedical and Diagnostic Sciences, University of Salamanca, Salamanca, Spain

**Keywords:** Older adult, Physical activity, Nutrition, Body composition, Quality of life

## Abstract

**Background:**

The increasing use of smartphones by older adults also increases their potential for improving different aspects of health in this population. Some studies have shown promising results in the improvement of cognitive performance through lifestyle modification. All this may have a broad impact on the quality of life and carrying out daily living activities. The objective of this study is to evaluate the effectiveness of combining the use of smartphone and smartband technology for 3 months with brief counseling on life habits, as opposed to providing counseling only, in increasing physical activity and improving adherence to the Mediterranean diet. Secondary objectives are to assess the effect of the intervention on body composition, quality of life, independence in daily living activities and cognitive performance.

**Methods:**

This study is a two-arm cluster-randomized trial that will be carried out in urban health centers in Spain. We will recruit 160 people aged between 65 and 80 without cardiovascular disease or cognitive impairment (score in the Mini-mental State Examination ≥24). On a visit to their center, intervention group participants will be instructed to use a smartphone application for a period of 3 months. This application integrates information on physical activity received from a fitness bracelet and self-reported information on the patient’s daily nutritional composition. The primary outcome will be the change in the number of steps measured by accelerometer. Secondary variables will be adherence to the Mediterranean diet, sitting time, body composition, quality of life, independence in daily living activities and cognitive performance. All variables will be measured at baseline and on the assessment visit after 3 months. A telephone follow-up will be carried out at 6 months to collect self-reported data regarding physical activity and adherence to the Mediterranean diet.

**Discussion:**

Preventive healthy aging programs should include health education with training in nutrition and lifestyles, while stressing the importance of and enhancing physical activity; the inclusion of new technologies can facilitate these goals. The EVIDENT-AGE study will incorporate a simple, accessible intervention with potential implementation in the care of older adults.

**Trial registration:**

ClinicalTrials.gov Identifier: NCT03574480. Date of trial Registration July 2, 2018.

## Background

The progressive aging of the population calls for the launch of programs aimed at achieving healthy aging so that people can maintain their autonomy for as long as possible. Active aging is defined by the WHO as “the process of optimizing opportunities for health, participation and security, in order to enhance the quality of life as people age” [1], which could be achieved through interventions or health promotion and prevention programs which include innovative and cost-effective strategies, such as the use of various digital health technologies [2].

### New technologies and strategies to help older adults modify their lifestyles

Mobile health, or mHealth, is a fundamental support tool for clinical practice and in particular for information gathering and communication between health care professionals and patients. Mhealth includes the use of mobile phones, patient monitoring devices and other wireless devices, and is used for both the prevention, diagnosis and treatment of chronic diseases and the promotion of healthy lifestyles [3–5]. One of the most recent reviews on this topic indicates that interventions using smartphones have managed to improve physical activity and adiposity markers, obtaining greater effectiveness by combining these interventions with individualized counseling [6]. Although the adoption of digital technology related to health among the elderly has been slow, this trend is being modified by the development of easier-to-use devices [7, 8]. It is estimated that 60% of older adults regularly use the Internet and 18% have a smartphone, computer or tablet [8]. In addition, 30% regularly search for health information online [7]. The routine integration of digital technology into the health management strategies of older people will increase as people become more digitally experienced [9]. In an intervention based on the use of pedometers carried out among the adult population which included people up to 75 years of age [10], the number of steps rose by approximately one-tenth and walking intensity was also increased. However, it has not been investigated whether the effect on behavioral changes among people over the age of 60 could be greater if multiple lifestyle changes were addressed at the same time.

### New technologies and strategies to help older adults reduce weight and modify body composition

Obesity affects more than 35% of people over 65 in developed countries, with figures doubling over the last 30 years [11] alongside increasing morbidity and mortality [12–15]. In addition, weight gain in this age group mainly involves an increase in adipose tissue, which is responsible for the complications associated with obesity [16, 17] and a decrease in muscle mass, leading to greater functional disability [18–20]. Action on obesity in the ≥65 s should take into account associated comorbidity and functional limitations [16, 21, 22]. The different strategies aimed at weight loss for older subjects have obtained controversial results [16, 17]. In most of the interventions performed in this age group, weight loss is associated with an increase in the loss of muscle and bone mass. [16, 23]. It is thus necessary to seek a balance, minimizing the negative effects while pursuing the advantages of reducing excess body weight [23]. Interventions involving new technologies have been carried out mostly among young people and the adult population, with very few involving subjects ≥65. In general, these interventions have yielded positive results, as reflected in several meta-analyses [6, 24–26] and numerous clinical trials [27–32]. On the other hand, most studies used small samples and did not assess whether weight loss was obtained at the expense of fat mass or lean mass.

### New technologies to help older adults improve quality of life and daily living activities

One of the goals of mHealth is to involve patients in the design and implementation of strategies to improve health and increase the general quality of life [33]. Health is closely linked to quality of life aspects, such as psychological well-being, autonomy, mobility, safety and social participation [34]. In turn, these aspects are affected by personal and environmental factors [35] which are conditioned by the state of health and which usually worsen with age [36]. Older people are interested in finding out about applications that could be useful in the realization of their daily activities [37], which suggests that such tools may help them in adapting to the environment and enable them to carry out daily living activities. Independence is imperative for older adults in order to avoid social exclusion [38], and in terms of control and choice, it is also of great importance when they access health services via mobile applications. Therefore, this type of device appears to perform an important service by promoting mobility and autonomy among older adults, which in turn can positively affect their quality of life [39].

### New technologies to help older adults improve cognitive performance

Increasing longevity has led to a growing prevalence of neurodegenerative diseases and ensuing cognitive deterioration and dementia. Recent studies suggest that maintaining a healthy lifestyle is associated with a lower risk of cognitive impairment [40, 41]. Indeed, it appears that maintaining certain eating patterns and healthy lifestyles, such as physical activity, could prevent it [42]. Thus, interventions focused on increasing physical activity and adherence to the Mediterranean diet, separately and in combination, not only reduce the risk of cognitive decline, but are also able to improve cognitive performance in healthy older people [43, 44]. Moreover, interest in the effect that the use of mHealth and new technologies in particular can have on cognitive performance is growing. Although more studies are needed in this area, existing results are promising. It has been proved that specific physical exercise interventions using mobile applications have beneficial health effects for healthy older adults and even patients with mild cognitive impairment and dementia; it has also been shown that they have no difficulty in adapting to the use of these new technologies [45, 46].

The use of electronic devices and smartphones by the older population has grown significantly, a fact which increases the potential for their use to improve different aspects of health for this age group. Most studies so far have been carried out with the young adult or middle-aged population; thus, despite their potential, the usefulness of new technologies has been little studied in older adults. There are some pilot studies noting the combined effect of these technologies alongside professional advice in lifestyle improvement, but the size of these studies does not adequately substantiate this relationship and its relevance in clinical terms. Results of the EVIDENT II study [47], which was carried out with adults, show that an intervention combining standardized counseling on diet and PA with the use of an app which incorporates personalized recommendations achieves beneficial results in terms of modifying anthropometric measurements among women. These results include a reduction in visceral adiposity. In addition, other studies have provided promising results in improving cognitive performance when maintaining a healthy lifestyle, even in people with mild cognitive impairment. All this may have a far-reaching impact on the quality of life and reduce cardiovascular risk factors.

### Hypothesis and objective

The main hypothesis of the EVIDENT-Age study is that lifestyle improvements among the over-65 population, achieved through the combined use of smartphones and smartbands, could have an influence in terms of improving body composition, quality of life, daily living activities and cognitive performance.

The objective of this study is to assess the effectiveness of the combined use of smartphone and smartband technology for 3 months alongside brief counseling on life habits, versus counseling alone, in increasing physical activity and improving adherence to the Mediterranean diet. As a secondary objective, we will assess the effect of the intervention on body composition, quality of life, daily living activities and cognitive performance.

## Methods/design

### Trial design

This study is a controlled and randomized parallel-group clinical trial, to be carried out in three health centers of the community of Castilla y León in Spain (La Alamedilla Health Centre, Garrido Sur Health Centre and Miguel Armijo Health Centre). The study has been registered at ClinicalTrials.gov Identifier: NCT03574480. Date of trial Registration July 2, 2018. The study was designed in accordance with the SPIRIT guidelines [48].

### Participants

Participants will be selected in the primary care surgeries of the participating health centers by consecutive sampling among those who meet the selection criteria. A member of the research staff will then give candidates the necessary information, after which they voluntarily sign the informed consent. In the case of older people who have difficulties reading and understanding consent properly, research staff will read them the information it contains and explain it.

The subjects will be informed of the objectives of the project and of the risks and benefits of the research to be carried out, including collection of samples. Confidentiality of participant data will be guaranteed at all times in accordance with the provisions of the Organic Law on the Protection of Personal Data (15/1999 of December 13, LOPD), and under the conditions established by Law 14/2007 of Spanish biomedical research.

### Inclusion criteria

Patients of both sexes treated in the health center surgeries, and aged between 65 and 80. Score equal to or greater than 24 points in the Mini Mental State Examination (MMSE).

### Exclusion criteria

People who cannot perform physical activity or follow a Mediterranean diet; coronary or cerebrovascular atherosclerotic disease, grade II or higher heart failure according to New York Heart Association (NYHA) criteria, moderate or severe chronic obstructive pulmonary disease, musculoskeletal disease limiting ambulation, advanced kidney or liver disease, severe mental illness, and cancer under treatment diagnosed in the previous 5 years or terminal.

### Sample size calculation

The sample size was estimated for the main variable of the study (Physical activity - steps/day). Assuming a standard deviation of 3847 steps/day [49], a power of 0.8 and an alpha risk of 0.05 in a bilateral test, 80 participants are necessary in each group to detect an increase of 1850 steps/day in the experimental group compared to the control group. This calculation was based on the estimate by Alonso-Dominguez et al. [50]. We assumed a dropout rate of 15% throughout the duration of the study (3 months).

### Randomization

**Fig. 1:**
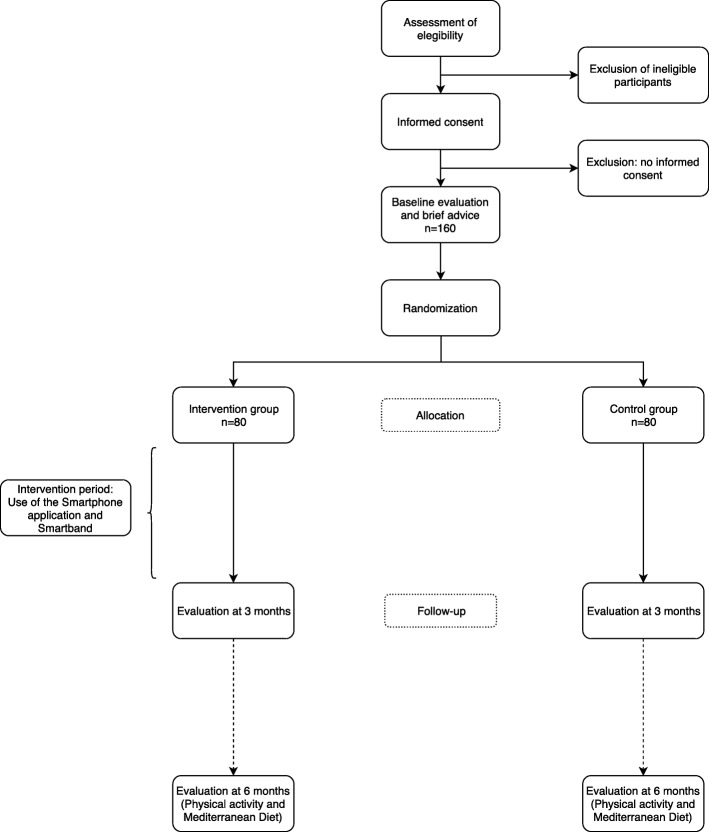
EVIDENT-Age study flow chart

Participants will be randomly assigned to the intervention (IG) or control group (CG) – Fig. 1. The allocation sequence will be generated by an independent researcher using Epidat 4.2 [51] and will remain secret until the participants are assigned to each group.

### Intervention

Common to both groups: before randomization, all participants (control and intervention), will receive nutritional counseling aimed at good compliance with the Mediterranean diet. Counseling will consist of a 10-min individual appointment in which the ideas behind the Mediterranean diet will be outlined, as well as the health benefits and evidence generated by this pattern of nutrition. Both groups will also receive brief counseling on physical activity aimed at complying with the current recommendations for both the general and the elderly population, and will be advised to perform at least 30 min of moderate activity for five days a week, or 20 min of vigorous activity three days a week. Participants will be provided with an informative leaflet at the end of the session.

**Fig. 2:**
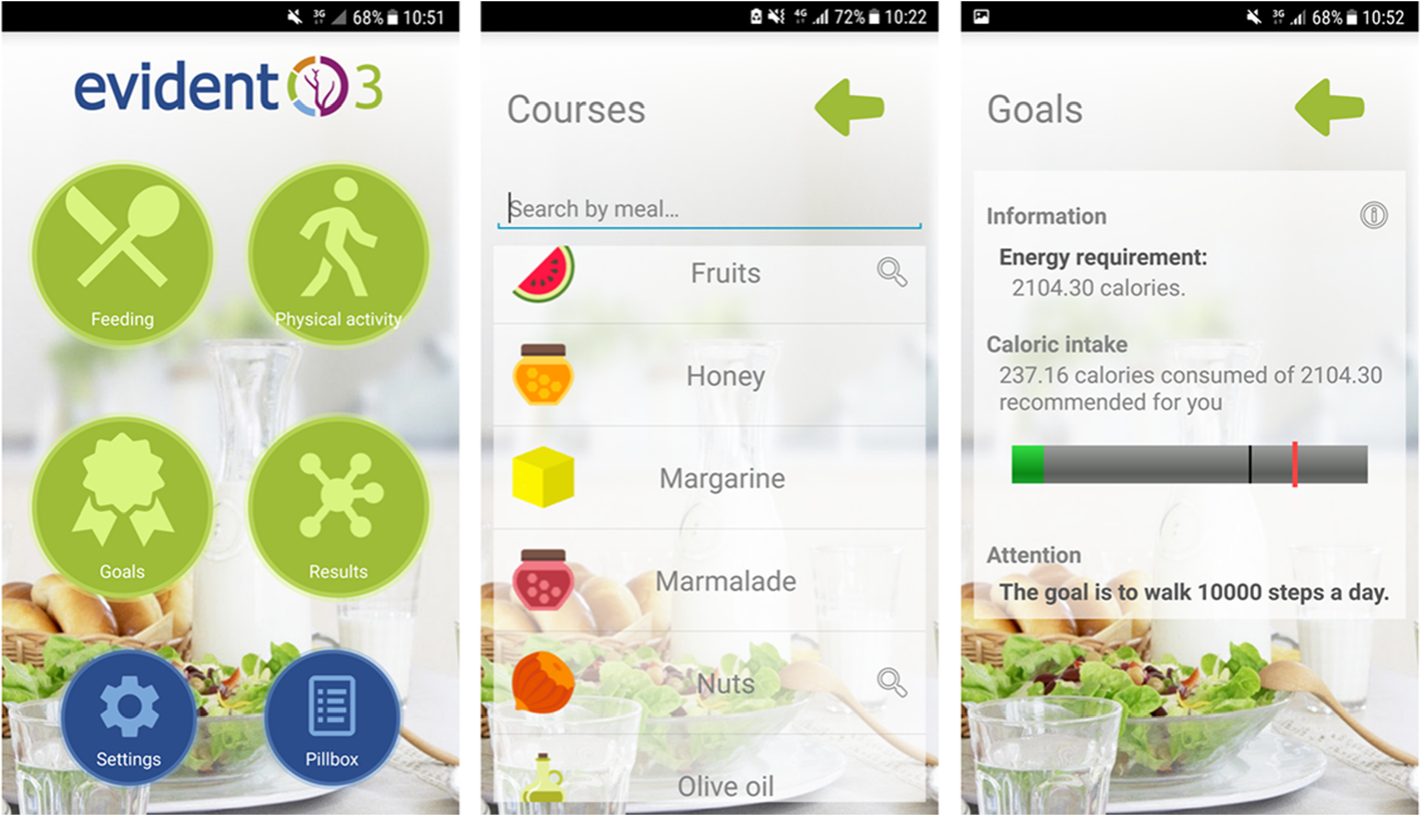
Screenshots of the EVIDENT smartphone application

Specific intervention in the experimental group: Participants in the intervention group will be issued with a smartphone (Samsung Galaxy J3) and a smartband (Xiaomi Miband S2) for the duration of intervention (3 months). During an approximately 15-min visit, each participant will be trained to use the device and the app developed for the EVIDENT 3 study [52] (Intellectual Property Registry No. 00/2017/2438), which is pre-installed on the smartphone. In the first part of this visit, the app will be configured with a series of data (age, sex, weight and height) which will allow the daily energy needs of each participant to be calculated. The app allows daily monitoring of food and physical activity by integrating the information collected by the smartband regarding physical activity (steps, calories and heart rate) and information related to food after the subject has logged food or dishes eaten each day, selected from the app menu –Fig. 2. At the end of the day, the application makes recommendations and a personalized plan to improve eating habits and physical activity over the following days. At the 3-month visit, the devices will be taken back.

### Data collection / outcomes

Each participant will undergo a baseline assessment and a follow-up evaluation at 3 months. Some variables (self-reported physical activity and adherence to the Mediterranean diet) will also be collected in a follow-up phone call at 6 months. The information generated by the app during the intervention period (physical activity and food) will also be analyzed. An overview of data collection is presented in Table 1.

**Table 1:** Timeline of measures

Measure	Baseline	3 months	6 months
Accelerometer	X	X	
International physical activity questionnaire (IPAQ)	X	X	X
Marshall questionnaire- sitting time	X	X	X
Mediterranean diet screener (MEDAS)	X	X	X
Body composition- impedanciometer	X	X	
Mini-Mental State Examination, the clock drawing test and categorial fluency	X	X	
Pfeffer questionnaire	X	X	
WHOQOL-AGE	X	X	
EuroQoL 5-D (EQ-5D)	X	X	

*IPAQ* International physical activity questionnaire, *MEDAS* Mediterranean diet adherence questionnaire, *WHOQOL-AGE* World health organization quality of life age questionnaire

### Primary outcome

#### Physical activity

The main physical activity variable will be the change in the amount and intensity of physical activity expressed in steps/day, measured by accelerometer. This device also calculates the total time spent daily in light, moderate and intense physical activity and the kilocalories used. Validated GT3X accelerometers will be used [53]. Participants will wear the accelerometer strapped to the right side of their waist with an elastic belt for seven consecutive days. Data will be recorded minute by minute. If the accelerometer records 10 consecutive zeros over the course of 10 min, the measurement will be considered null. The intensity of physical activity (low, moderate or high) will be determined according to the cut points proposed by Freedson [54]. Physical activity will also be measured by self-report using the International Physical Activity Questionnaire (IPAQ). The short version will be used, which is validated in Spanish and assesses physical activity over the last 7 days [55]. This questionnaire classifies adult populations by their level of activity (low, moderate and high) and differentiates three types of activity: walking, moderate activity and vigorous activity. The physical activity dose will be estimated in METs/minute/week. An active person is considered to be someone who performs at least 30 min of moderate activity, five days a week, or at least 20 min of vigorous activity, three days a week, or reaches 450 METs/minute/week.

### Secondary outcomes

#### Sedentarism

The number of daily hours spent sitting will be collected using the questionnaire developed by Marshall et al. [56], which evaluates the hours that the individual remains seated while working, traveling and during leisure activities involving screens (television, computer, tablets/smartphone). The questionnaire differentiates between work and non-work days.

#### Dietary pattern

The change in adherence to the Mediterranean diet (MD) will be measured by the total scores (baseline and final) on the Mediterranean diet adherence questionnaire. This questionnaire, validated in Spain, has been used in the PREDIMED study [57] and asks 14 questions regarding MD compliance. Each question is scored with zero or one point. One point is given for using olive oil as the main fat for cooking, preferring white meat to red meat, daily consumption of four or more tablespoons (one tablespoon = 13.5 g) of olive oil (including oil used for frying, dressing salads, etc.), two or more servings of vegetables, three or more pieces of fruit, less than one serving of red meat or sausage, less than one serving of animal fat, less than one cup (one cup = 100 ml) of carbonated and/or sugary drinks; and also for weekly intake of seven or more glasses of wine, three or more servings of legumes, three or more servings of fish, two items or fewer of shop-bought pastries, three or more servings of nuts, two or more helpings of sofrito, (traditional sauce made with tomato, garlic, onion or leeks, sautéed with olive oil). The final score ranges from zero to fourteen points, with nine or more points representing good adherence to the Mediterranean diet.

#### Body composition

This will be measured by InBody 230 [58], which monitors body weight, muscle-skeletal mass, fat mass, total body water, fat-free mass, percentage of body fat, waist-hip ratio, basal metabolism, muscle-fat control, in addition to performing a segmental analysis of lean and fat mass. Height will be measured using a portable system (Seca 222), with the average of two measurements being recorded.

#### Cognitive performance

General cognitive capacity will be assessed by applying the Spanish validation of the Mini-Mental State Examination (MMSE) [59]. This test allows the performance of different cognitive functions to be tracked: temporo-spatial orientation, attention, learning and memory, executive functions, language and visuoconstructive skills. The score can range from 0 to 30 points, with greater than 24 points considered to represent normal cognitive performance. In addition, the clock test will be applied [60] to assess visuoconstructive skills, executive functions and language. Finally, verbal fluency ability will be measured by a categorical fluency test in which animals are listed for one minute [61].

#### Activities of daily living

The level of functionality will be assessed with the Functional Activity Questionnaire (FAQ) that was designed by Pfeffer and is based on a previous study by the same author [62, 63]. It is a screening scale for mild cases of dementia, with questions which assess the ability to carry out complex social activities, the so-called instrumental activities of daily living. It asks about the following 11 items: handling money, making purchases, preparing tea or coffee and switching off the appliance, preparing food, keeping up with community events, understanding and discussing radio and television news, reading magazines and books, remembering appointments and important dates, medication management, traveling alone outside the neighborhood, greeting friends and going outside alone safely. Each item scores between 0 (normal) and 3 (totally dependent).

#### Quality of life

This will be assessed by a modified version of the World Health Organization quality of life instrument called WHOQOL-AGE that has been specially adapted for the elderly population. This short version contains 13 of the 100 questions in the original version and has been validated in populations older than 50 years [64]. The results can range from 0 (lower quality of life) to 100 (higher quality of life). Similarly, health-related quality of life will also be evaluated with the EuroQol 5D questionnaire (EQ-5D). We will use an adapted version of this questionnaire which has been validated for the Spanish population [65]. This questionnaire comprises three elements: the individual’s self-rated state of health assessment in levels of severity by dimension (mobility, self-care, usual activities, pain/discomfort and anxiety/depression), assessment of his or her state of health on a visual analogue scale, and finally an index of social values ​​obtained for each health state generated by the instrument.

#### Other variables

Motivation for change, as described by Prochaska and Diclemente, will be assessed at baseline, classified into the different stages of pre-contemplation, contemplation, determination, action, maintenance and relapse. With intervention group participants, app adherence will be measured by the number of logs and days on the device.

### Data collection procedure, data management and monitoring

Data collection at baseline and follow-up visits at 3 and 6 months will be carried out by a specifically trained nurse. The brief counseling session after baseline assessment will be carried out by another nurse, different from the one responsible for data collection. Finally, a third member of the research team, who was not involved in either data collection or brief counseling, will be in charge of carrying out the intervention visit. Each participant will have a unique identification code within the study. All measurements will be compiled in a data collection notebook and kept in a secure place which will remain closed inside the health center. A database will be created in SPSS to which only research team members and people involved in the statistical analyses will have access. The principal investigator or a person designated for the purpose will perform weekly study monitoring covering patient inclusion, database cleaning and debugging, and adapting procedures to the protocol.

### Blinding

Given the nature of the intervention itself, participants cannot be blinded. However, strategies will be in place to attain the highest level of blinding possible. The researcher making the visit to the intervention group to explain the app will be different from the nurse responsible for taking the measurements and the person in charge of carrying out brief counseling after the baseline evaluation visit. In addition, the researcher responsible for the statistical analysis will not know the group to which the participants belong. To prevent contamination between groups, no additional counseling or reinforcement will be made during the 3-month evaluation visit. In addition, the app will not be available for download until the study is completed.

### Analysis plan

#### General analysis

Results will be expressed by means and standard deviations for quantitative variables, or by frequency distributions and percentages in the case of qualitative ones. The normality of variables will be tested by means of the Kolmogorov-Smirnov test. When a normal distribution cannot be assumed, we will use the corresponding nonparametric tests. The chi-square test or Fisher’s exact test will be applied to analyze the association between independent qualitative variables. Using the Student’s t-test or the Mann-Whitney U test, the means between the two groups will be compared. The relationship between quantitative variables will be analyzed with the Pearson or Spearman correlation coefficients.

Analysis of the results for the main variable and the secondary variables will be carried out by intention-to-treat. In addition, app adherence will be the subject of a secondary analysis, as described in the results of the EVIDENT II study [66] (< 60 days and > 60 days), and other relevant subgroups will also be analyzed in terms of their physical activity or previous adherence to the Mediterranean diet.

### Analysis of the effect of the intervention on primary (steps/day) and secondary outcomes

To analyze the changes at 3 and 6 months’ post-baseline in the primary (physical activity - steps/day) and the secondary outcome within the same group, the Student’s t-test or the Wilcoxon test will be used for paired data. The McNemar test will be used for dichotomous variables.

The intervention effect will be analyzed by comparing the changes between IG and CG in blood pressure and secondary variables by using ANCOVA and adjusting for possible confounders. The intervention effect over the period studied will be measured by a repeated measures analysis.

### Analysis by subgroups

The intervention effect could be affected by age, sex, educational level and adherence to the application during the study, as well as motivation and lifestyles at the baseline assessment. The same analyses described above will be performed for each of the aforementioned subgroups.

### Secondary analyses

Multiple multivariate regression analysis will be performed to identify the variables with the greatest influence on physical activity changes and the secondary variables analyzed.

All analyses will be carried out with SPSS version 23.0 (IBM Corporation, Armonk, NY, USA) and an alpha risk of 0.05 will be assumed as the limit for statistical significance.

## Discussion

Physical inactivity in people over 65 across Europe [67] makes it necessary to develop strategies and interventions aimed at healthy aging by maintaining older adult health, autonomy and independence for as long as possible. The promotion of a general framework to guide the design of healthy aging programs from a preventive approach which considers the essential aspects involved in aging would therefore be welcome. Such programs should include health education with training in nutrition and lifestyles, while focusing on and enhancing physical activity [68]. The promotion research on the aging process and rigorous assessment of all aspects of healthy living are necessary to achieve these objectives.

It has been proven that increased physical activity and healthy eating has health implications for the over-65 s [69]. This fact, alongside the unceasing incorporation of information technologies into the daily lives of older adults and their use as tools to adopt and maintain healthier lifestyles, makes it necessary to design simple and effective applications aimed at this population group. The novelty of the EVIDENT-Age study lies in the combination of brief lifestyle counseling with the use of a smartphone application. The effect of this application has been studied on the health of the general population, the overweight or obese population and the population of diabetes mellitus type 2 sufferers [50, 52, 70]. We have modified the application to take into account the characteristics of the study population; thus, for example, font size has been increased and the way the content is presented has been modified. In addition, the application has features which have been described as essential for the elderly, such as continuous feedback for the app user, target setting and rewards, as well as the inclusion of social factors. Nevertheless, it is not clear which of these components may be more effective in achieving the objectives [71]. The effects of this intervention can be quickly transferred to clinical practice or included in health education programs for older adults. The results of this work will be communicated to participants and sponsors, as well as to health professionals and the general public. The study will result in several different publications in peer reviewed journals.

## Abbreviations

CG: Control group
IG: Intervention group
MD: Mediterranean diet
Mhealth: Mobile health
